# Investigation of the 3D Printing Process Utilizing a Heterophase System

**DOI:** 10.3390/gels9070566

**Published:** 2023-07-12

**Authors:** Natalia Menshutina, Andrey Abramov, Maria Okisheva, Pavel Tsygankov

**Affiliations:** Department of Chemical and Pharmaceutical Engineering, Mendeleev University of Chemical Technology of Russia, Miusskaya pl. 9, 125047 Moscow, Russia; chemcom@muctr.ru (N.M.); abramovandrey516@gmail.com (A.A.); okisheva.m.k@muctr.ru (M.O.)

**Keywords:** 3D printing, heterophase system, gelatin, sodium alginate

## Abstract

Direct ink writing (DIW) requires careful selection of ink composition with specific rheological properties, and it has limitations, such as the inability to create overhanging parts or branched geometries. This study presents an investigation into enhancing the 3D printing process through the use of a heterophase system, aiming to overcome these limitations. A modification was carried out in the 3D printer construction, involving adjustments to the structural elements responsible for the extrusion device’s movement. Additionally, a method for obtaining a heterophase system based on gelatin microparticles was developed to enable the 3D printing process with the upgraded printer. The structure and rheological properties of the heterophase system, varying in gelatin concentration, were thoroughly examined. The material’s viscosity ranged from 5.4 to 32.8 kPa·s, exhibiting thixotropic properties, pseudoplastic behavior, and long-term stability at 20 °C. The developed 3D printing technology was successfully implemented using a heterophase system based on different gelatin concentrations. The highest product quality was achieved with a heterophase system consisting of 4.5 wt.% gelatin, which exhibited a viscosity of 22.4 kPa·s, enabling the production of products without spreading or compromising geometrical integrity.

## 1. Introduction

Additive technologies are considered to be one of the most promising methods for producing complex geometric products with customized properties for specific applications [1]. Currently, there is a growing interest among researchers in developing three-dimensional printing techniques using viscous materials, especially polymer solutions [2,3]. Among the various methods for 3D printing with viscous materials, extrusion-based techniques stand out. These methods involve the extrusion of ink and enable layer-by-layer formation of the desired product [4,5]. Depending on the presence of an additional supporting matrix during layer-by-layer formation, two distinct approaches are recognized: direct ink writing and three-dimensional printing using a heterophase system.

Direct ink writing involves the extrusion of viscous materials directly onto the printing surface. To ensure successful implementation of this technology, careful selection of ink composition with specific rheological properties is required. It should prevent spreading during layer-by-layer object formation while maintaining proper extrusion capability [6,7]. However, direct ink writing has limitations, as it cannot produce products with overhanging parts, hollow structures, or complex geometries due to insufficient mechanical strength during layer-by-layer formation, leading to potential printing defects [5].

Expansion of the application areas of 3D printing with viscous materials can be achieved by utilizing a heterophase system. This approach involves extruding ink into a volume containing microparticles suspended in a liquid medium. The high viscosity of the system provides support for complex geometries during layer-by-layer formation. The heterophase system can include components that facilitate crosslinking of the extruded ink [8]. This strategy reduces the requirements for ink rheological properties and enables the production of defect-free products with diverse geometries, unrestricted by gravity [9,10]. Consequently, this technology is versatile and compatible with materials of varying viscosities, extending the potential applications of 3D printing in regenerative medicine [11].

A properly formulated heterophase system must possess specific rheological properties [12]. Particularly, it should exhibit pseudoplastic flow behavior characterized by a viscosity decrease under shear stresses. This property allows smooth movement of the 3D printer nozzle within the heterophase system during layer-by-layer object formation [13]. The system should also demonstrate thixotropic properties, which refer to its ability to restore viscosity after shear stress removal. These properties are crucial for providing structural support to the printed product and preventing ink spreading during 3D printing [12,14]. Additionally, the destabilizing external forces that occur during the printing process must be taken into account to ensure successful implementation of 3D printing with a heterophase system [15]. Four types of destabilizing forces or instabilities are recognized:

- Reynolds instability (emergence of turbulent flows in a heterophase system at a high speed of the extruder nozzle) [14];
- The occurrence of air pockets (formation of air pockets due to the high speed of the extruder nozzle, insufficiently pronounced thixotropic properties and low pseudoplasticity degree of the heterophase system) [14,16];
- Archimedean instability (surfacing of extruded ink during the printing process due to a mismatch between the densities of the ink and the heterophase system) [17,18];
- Rayleigh–Plateau instability (breaking of the extruded ink flow due to the high surface tension of the heterophase system) [19,20].

Overcoming the presented types of instability is achieved with a detailed study of the rheological features, composition and structure of the heterophase system.

Currently, there are a number of studies that provide details on the heterophase system composition development, as well as the 3D printing process using the developed system. Examples of heterophase systems are listed in Table 1.

This table shows the range of applications of heterophase systems that highlight the versatility and customizability of 3D printing using supporting system. This approach has been shown to work with numerous materials, such as carbopol, pluronic F-127, xanthan gum methacrylate and agarose. However, gelatin is the most promising heterophase system material. This is due to a number of physicochemical properties of gelatin. Firstly, its gelation occurs due to a temperature change. This makes it possible to extract an object with complex geometry from a heterophase system due to a temperature increase, which leads to a decrease in the viscosity of the gelatin solution. Secondly, gelatin microparticles provide the required rheological properties necessary for the 3D printing process using a heterophase system.

This article presents the stages of development of 3D printing technology using the heterophase system, including modification of the 3D printer design, obtaining ink based on sodium alginate, and choosing the composition of the heterophase system based on gelatin. Using the developed 3D printing technology, the printing process was implemented with ink, the viscosity of which is less than 100 Pa∙s.

## 2. Results and Discussion

### 2.1. Investigation into the Composition and Properties of a Heterophase System

**Figure 1:**
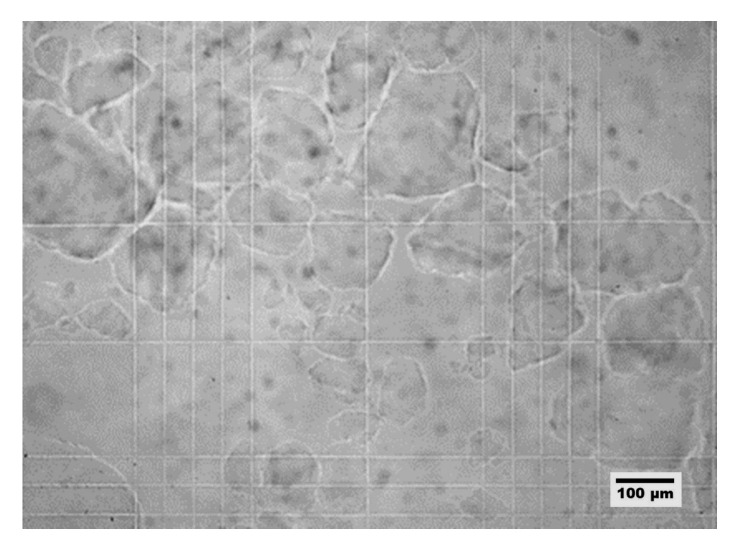
Optical microscopy images of heterophase system microparticles based on gelatin concentration 4 wt.%.

The developed method made it possible to create a heterophase system composed of gelatin microparticles at various concentrations suspended in a crosslinking agent solution. Optical microscopy and a Goryaev camera were employed to examine the impact of gelatin concentration on particle size and morphology. Figure 1 showcases the visualization of microparticles within the heterophase system, using gelatin with a concentration of 4 wt.%. It is worth noting that the concentration of gelatin in the solution does not affect the morphology and particle size.

Based on the data acquired, it is evident that the dispersion carried out at these parameters leads to the formation of gelatin microparticles of irregular shape.

**Figure 2:**
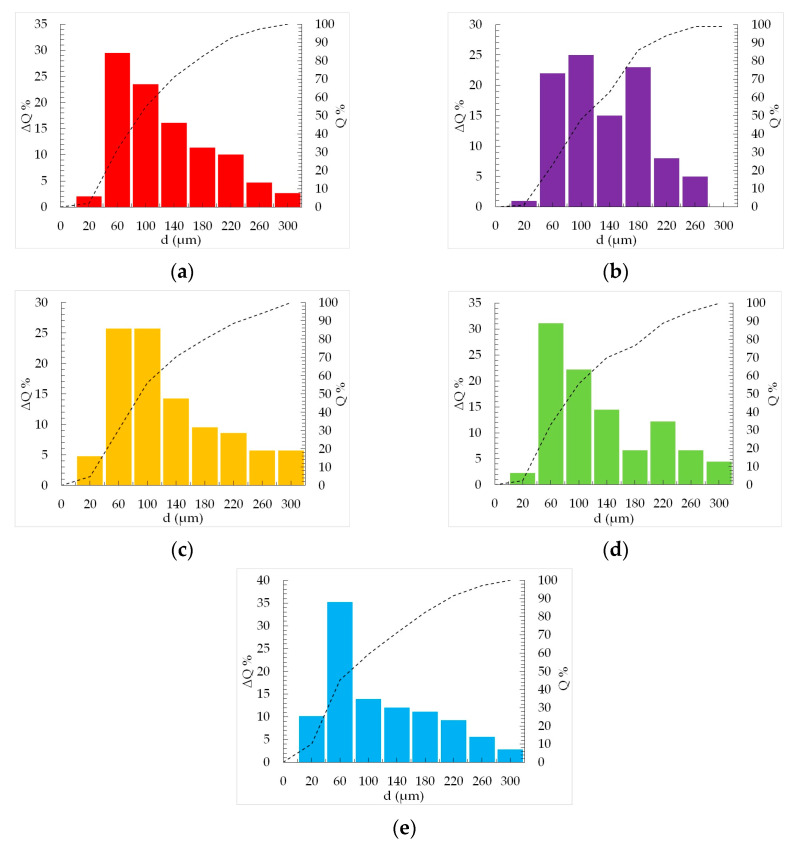
Particle size distribution analysis of a heterophase system based on different gelatin concentrations (**a**) 3 wt.%, (**b**) 3,5 wt% (**c**) 4 wt% (**d**) 4,5 wt% (**e**) 5 wt%.

To gain a comprehensive understanding of the particle size characteristics, an in-depth analysis was performed, involving the construction of integral and differential particle size distribution curves. These curves were derived by closely examining optical microscope images obtained for each concentration of gelatin studied. The outcomes of the analyses are presented in Figure 2.

Based on the obtained results, it has been determined that the concentration of gelatin does not have a significant impact on the average particle diameter within the heterophase system. Across all studied concentrations, the average particle diameter ranges from 60 to 100 μm. This observation highlights the stability of particle size, regardless of gelatin concentration.

When implementing a 3D printing process using a heterophase system, special attention is dedicated to particle size and particle size distribution. These factors play a crucial role in the realization of the 3D printing process, ultimately influencing the quality of the final product. Based on the data presented, it can be concluded that the gelatin concentration does not exert a significant influence on particle size. Consequently, this factor can be considered as non-destabilizing in the context of three-dimensional printing utilizing a heterophase system.

To attain the desired quality of the final product, it is essential to ensure that the heterophase system possesses specific rheological properties. The developed composition should exhibit pseudoplastic and thixotropic behavior while maintaining a yield strength that aligns with the rheological characteristics of the ink. These rheological features are crucial for achieving optimal printing results and ensuring the integrity of the printed objects.

**Figure 3:**
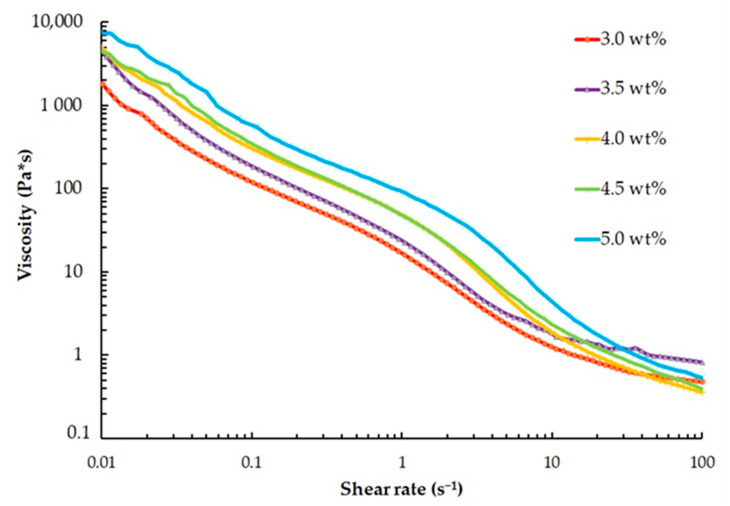
Viscosity as a function of shear rate for the heterophase system based on different gelatin concentrations (logarithmic axes).

The rheological properties of the heterophase system were evaluated using an AntonPaar SmartPave 102e rotational rheometer equipped with a 50 mm diameter plane-cone measuring unit. To investigate the influence of gelatin concentration on the material’s pseudoplastic behavior, measurements were conducted with a shear rate ranging from 0.01 s^−1^ to 100 s^−1^. The obtained research data were used to construct viscosity curves, as shown in Figure 3.

The viscosity of all solutions exhibits a decreasing trend with increasing shear rate, indicating pseudoplastic behavior. To determine the viscosity of the solutions at the lowest shear rate, a comprehensive study was conducted at a constant shear rate of 0.01 s^−1^. The results of this study are summarized in Table 2, providing valuable insights into the viscosity values at this specific shear rate for each solution.

An increase in concentration of gelatin leads to a significant elevation in the viscosity of the investigated compositions of the heterophase system, with higher viscosity values corresponding to increased yield strength.

The 3D printing process involves the sequential deposition of materials layer-by-layer along a predetermined path. Consequently, the structure of the heterophase system undergoes multiple impacts during printing. Therefore, it is crucial to evaluate the viscosity recovery of the materials after repeated exposure to shear stresses when selecting an appropriate composition.

**Figure 4:**
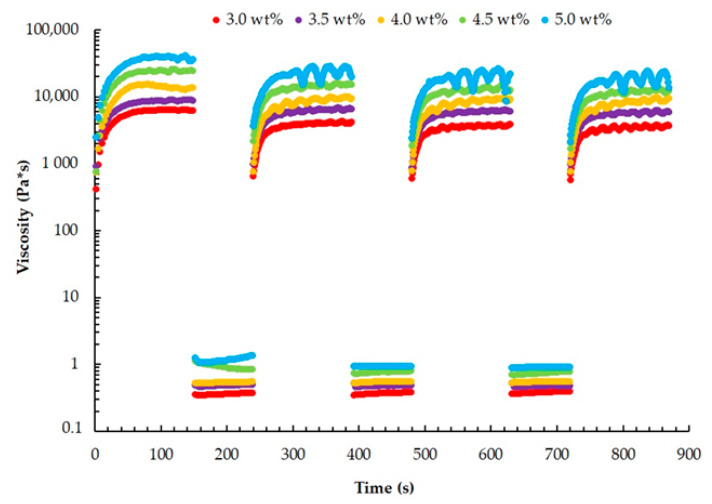
Viscosity as a function of time for the thixotropic test of heterophase system based on different concentrations of gelatin (logarithmic axes). Measurement intervals: (1) measurement at the shear rate 0.01 s^−1^ (0–150 s); (2) measurement at the shear rate 100 s^−1^ (150–240 s); (3) measurement at the shear rate 0.01 s^−1^ (240–390 s); (4) measurement at the shear rate 100 s^−1^ (390–480 s); (5) measurement at the shear rate 0.01 s^−1^ (480–630 s); (6) measurement at the shear rate 100 s^−1^ (630–720 s); (7) measurement at the shear rate 0.01 s^−1^ (720–870 s).

In that way, the presence of thixotropic properties is an essential requirement for utilizing the gelatin microparticles obtained through the developed method as a heterophase system. To assess these properties, a comprehensive study was conducted, involving several measurement intervals: (1) viscosity measurement at the minimum shear rate, (2) viscosity measurement at the maximum shear rate to disrupt the material’s structure, and (3) viscosity measurement at the minimum shear rate to determine the system’s recovery time (Figure 4).

The research results have shown the presence of thixotropic properties in all the analyzed solutions. This characteristic plays a crucial role in maintaining the structural integrity of the product during its layer-by-layer formation within the heterophase system. After passing through the extruder nozzle, the initial viscosity of the system is rapidly restored, ensuring proper support and stability ink.

**Figure 5:**
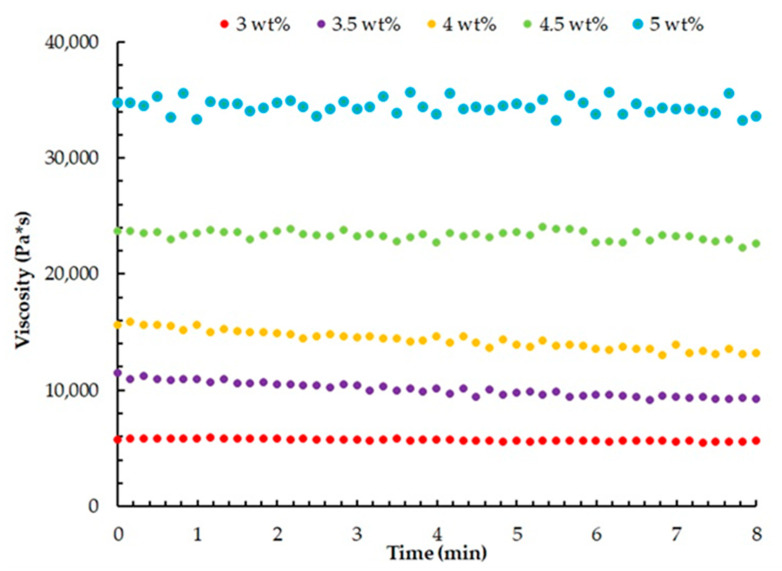
Time-dependent viscosity variation of the heterophase system at a constant temperature of 20 °C for different gelatin concentrations.

To evaluate the long-term stability of the studied materials, the tests were conducted at a controlled temperature of 20 °C. The experimental data, depicted in Figure 5, provide insights into the materials’ ability to maintain their properties and performance under typical operating conditions.

At all the investigated gelatin concentrations and a temperature of 20 °C, there was no decrease in the viscosity of the heterophase systems, indicating the structural stability of the system. This characteristic is particularly significant as it allows for the implementation of the 3D printing process at room temperature without any alteration to the properties of the heterophase system.

### 2.2. Investigation of the Properties of Sodium Alginate Ink

Viscous ink based on sodium alginate has garnered considerable attention due to its wide-ranging potential applications. The purpose of this study was to investigate the properties of sodium alginate ink and assess its suitability for 3D printing.

In a previous study [31], it was demonstrated that direct gel printing requires ink with high viscosity at low shear rates. This high viscosity enables layer-by-layer formation of three-dimensional objects without undesired spreading. The authors of [31] identified a partially cross-linked polymer with a viscosity of 1680.9 Pa·s, which meets the accuracy requirements for direct gel printing. However, the incorporation of a heterophase system in the developed technology significantly relaxes the rheological property demands of the ink.

The rheological properties of sodium alginate ink were evaluated using an AntonPaar SmartPave102e rotational rheometer. Dynamic viscosity of the solutions was determined by measuring at a constant minimum shear rate of 0.01 s^−1^. The dependence of viscosity on the concentration of sodium alginate solution is presented in Table 3.

The viscosity of the sodium alginate solution is influenced by both the concentration of the polymer in the solution and the length of the polymer molecule. In this experiment, sodium alginate of the same brand was used, ensuring that the effect of polymer length on the rheological properties is eliminated. Increasing the concentration of sodium alginate results in higher viscosity, attributed to the increased entanglement of polymer chains within the solution.

**Figure 6:**
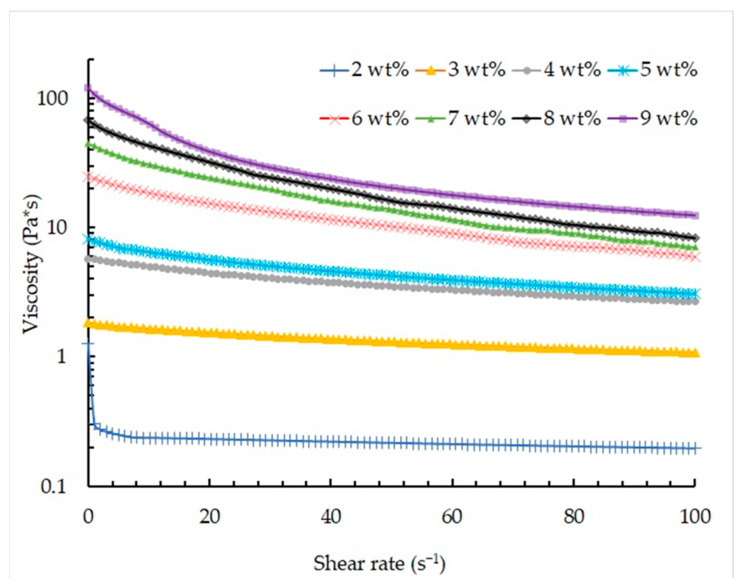
Viscosity as a function of shear rate for the viscous ink based on different sodium alginate concentration (logarithmic axes).

To investigate the pseudoplastic behavior of the solutions, viscosity curves were generated by progressively increasing the shear rate from 0.01 s^−1^ to 100 s^−1^. Figure 6 illustrates the viscosity changes observed in the studied solutions as the shear rate increases.

In this study, Equation (1) was employed to determine the power index values, aiming to assess the influence of sodium alginate concentration on the degree of pseudoplasticity of the materials. The coefficients for the equation were selected through multivariate optimization using the Powell method. The relative error was chosen as the optimality criterion, with a range of 3% to 7%.

τ = k∙γ^n^, (1)

where, τ—shear stress, Pa; γ—shear rate, s^−1^; k—consistency coefficient, Pa∙s^n^; and n—power index.

**Figure 7:**
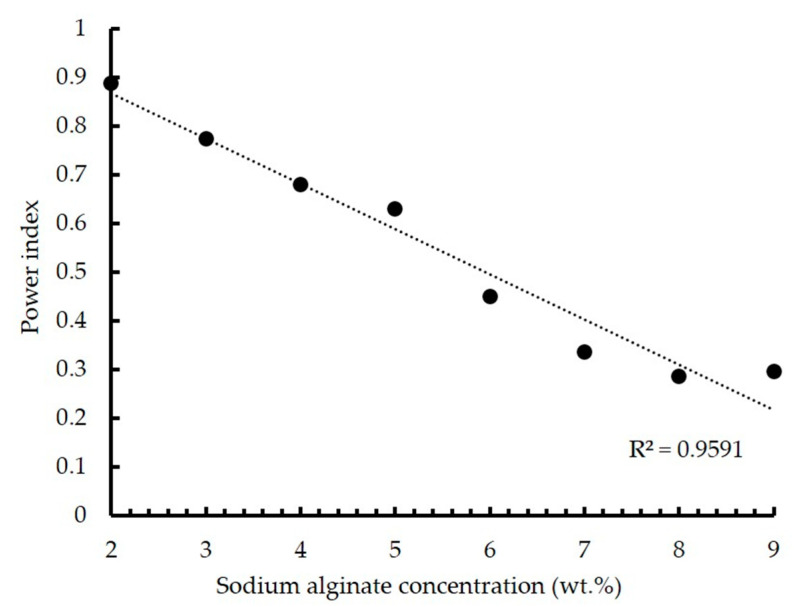
Dependence of the power index on the sodium alginate concentration.

Figure 7 illustrates the relationship between the power index and the concentration of sodium alginate in the solution.

According to the presented graph, it can be concluded that all the studied solutions have pseudoplastic behavior, which is also confirmed by the analysis of the graphs shown in Figure 6. An increase in sodium alginate concentration leads to an increase in the degree of pseudoplasticity, which is due to an increase in the number of polymer chains in the solution.

Thus, all analyzed solutions can be used to implement the 3D printing process using a heterophase system. However, an increase in sodium alginate concentration contributes to a decrease in the accuracy of the printing process due to clogging of the extruder nozzle due to high viscosity. In addition, the high viscosity of solutions limits the possibility of developing composite “ink” compositions that ensure biocompatibility and cell adhesion on the surface and in the internal structure of the resulting products. Based on the studies, a solution with sodium alginate concentration of 2 wt.%, characterized by a viscosity of 0.8 Pa∙s and a power index value of 0.9, was chosen as the ink composition for the developed technology of the three-dimensional printing using a heterophase system.

### 2.3. Investigation of the 3D Printing Process Using a Heterophase System

To investigate the influence of the composition and properties of the heterophase system, as well as the sodium alginate-based ink, on the 3D printing process, the developed technology was utilized to successfully fabricate products with intricate geometries.

**Figure 8:**
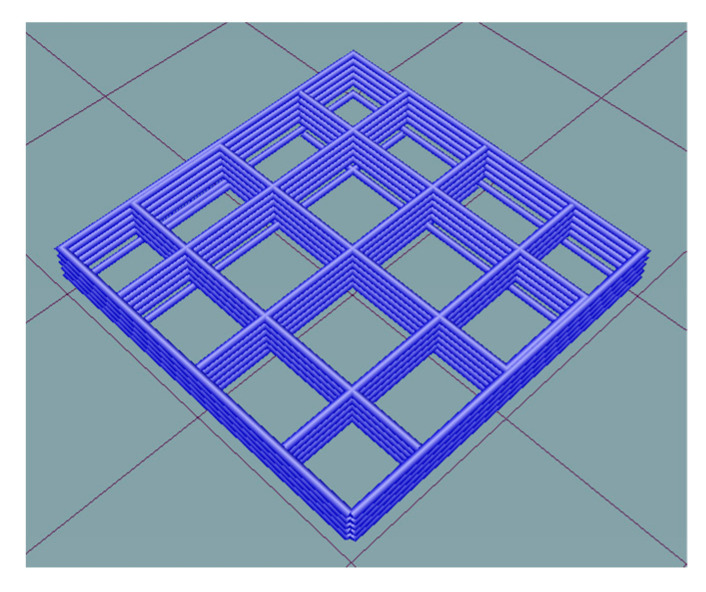
Test model for evaluating print accuracy.

The 3D printing process was controlled using specialized RepetierHost software, which provided precise instructions for the printing procedure. A test model in the form of a 20 mm × 20 mm × 3 mm plate was designed using computer-aided design systems. The internal structure of the model was filled with a grid pattern at a density of 15%, using the RepetierHost slicer, enabling the evaluation of printing accuracy (Figure 8).

**Figure 9:**
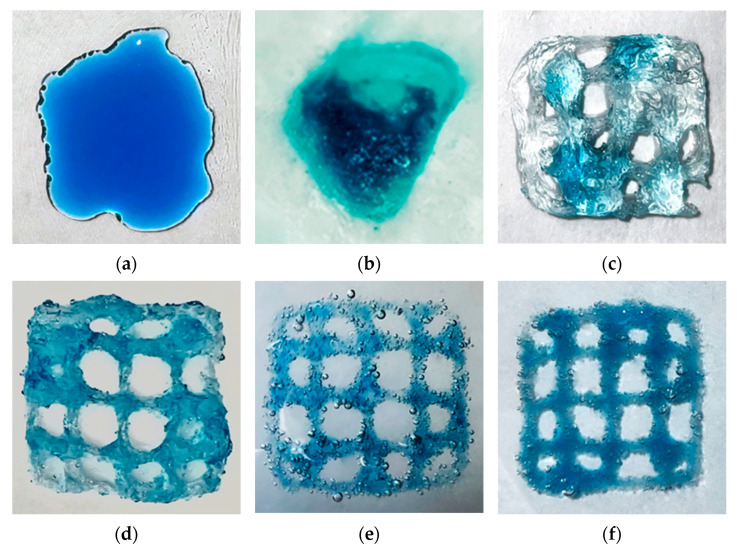
The result of the 3D printing process using a heterophase system containing various concentrations of gelatin: (**a**) 0 wt.%; (**b**) 3.0 wt.%; (**c**) 3.5 wt%; (**d**) 4.0 wt.%; (**e**) 4.5 wt.%; (**f**) 5.0 wt.%.

To study the influence of the structure and composition of the heterophase system on the 3D printing process, the following process parameters were chosen: layer thickness 0.6 mm, extruder nozzle speed 6 mm/s, extruder piston speed 0.01 mm/s, and printing temperature 20 °C (Figure 9).

Based on the provided images, it is evident that the viscosity of the heterophase system plays a crucial role in the 3D printing process. When using gelatin concentrations ranging from 0 to 4 wt.%, the viscosity of the system is inadequate. This leads to material spreading during printing, resulting in deviations from the intended geometry of the printed product. Conversely, gelatin concentration of 5 wt.% results in excessively high viscosity, leading to material sagging and distortion of the product’s geometry. This can be attributed to the increased yield strength, making it difficult to move the printing device nozzle and causing the formation of air pockets. The optimal print quality was achieved with a heterophase system based on a gelatin concentration of 4.5 wt.%.

Furthermore, the accuracy index for the obtained 3D models was calculated using Equation (2):

(2) $$\%\mathrm{Accuracy}=\left[\frac{1}{n}\sum_{i=1}^{n}\left(1-\frac{\left|A_{i}-A\right|}{A}\right)\right]\times 100\%,$$

where, *A_i_* is measured channel area, *A* is design area and *n* represents total number of channel in a printed construct.

In Table 4, the calculated accuracy of the obtained 3D printed models is presented.

The presented data confirm the high accuracy of the final products in the implementation of the 3D printing process using the heterophase system based on a gelatin concentration of 4.5 wt.%. Thus, in the implementation of the developed 3D printing technology using viscous sodium alginate ink with a concentration of 2 wt.%, this concentration of the heterophase system is the most suitable. However, to enhance the accuracy of the 3D printing process, further optimization of the printing parameters is necessary, including the speed of the printing device movement, the feed rate of the gelatinous materials, and the layer thickness. Additionally, variations to the diameter of the extruder nozzle outlet can be performed to optimize the printing process.

## 3. Conclusions

In this study, a 3D printing technology using the heterophase system was developed and implemented. To facilitate the printing process, the 3D printer design presented in [31,32] was upgraded, incorporating newly developed structural components produced via liquid crystal stereolithography.

A method for obtaining a heterophase system based on gelatin microparticles was developed to enable the 3D printing process using the modernized 3D printer design. Research findings indicated that the gelatin concentration had little to no effect on the morphology and size of the microparticles. Rheological studies of the heterophase system, considering various gelatin concentrations, revealed the presence of desirable thixotropic properties and pseudoplastic behavior, which are essential for the successful implementation of the developed technology. The viscosity of the obtained materials ranged from 5.4 to 32.8 kPa·s. Furthermore, all tested compositions of the heterophase system exhibited stability at room temperature (20 °C), ensuring prolonged printing without complications.

Investigations into the influence of gelatin concentration on the 3D printing process demonstrated that the best-quality products were achieved using the heterophase system composed of 4.5 wt.% gelatin. This specific concentration provided the necessary viscosity (22.4 kPa·s), enabling the production of printed objects without spreading, distortion, or geometry destruction.

## 4. Materials and Methods

### 4.1. Materials

Gelatin (CAS Number: 9000-70-8, Merck KGaA, Darmstadt, Germany) and anhydrous calcium chloride (RusChem, Moscow, Russia) were used to obtain a heterophase system. Alginic acid sodium salt (CAS Number: 9005-38-3, Sigma-Aldrich, St. Louis, MO, USA) was used as an ink precursor.

### 4.2. Development of the 3D Printer Construction

**Figure 10:**
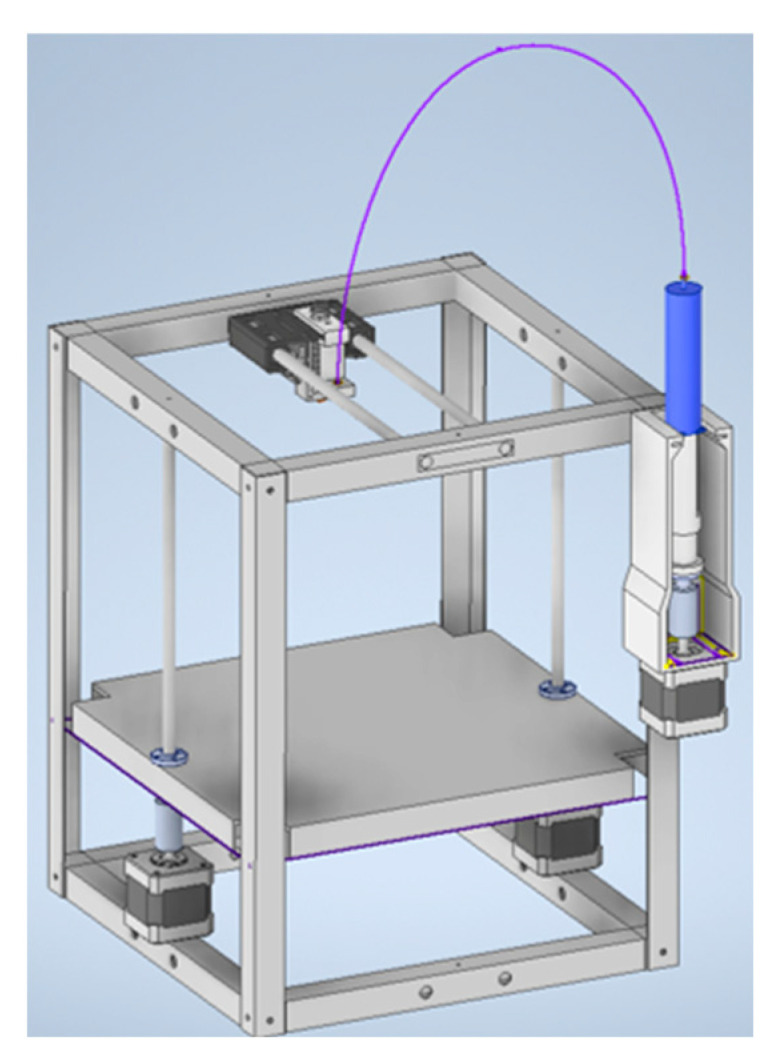
Construction of the 3D printer for the direct ink writing [31].

To implement the 3D printing process using a heterophase system, a modification of the device design, presented in [31,32] (Figure 10), was carried out.

**Figure 11:**
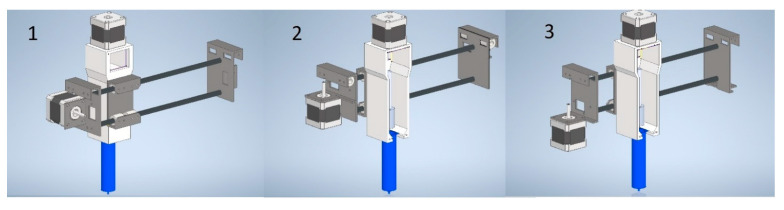
Design options of structural elements and the location of the stepper motor on them.

In order to modify the arrangement of the movable elements of the 3D printer, a number of models were developed in the computer-aided design system to change the orientation of the extruder carriage. A viscous ink extruder, proposed in [31,32] (Figure 11), was chosen as a punching device.

The determining factor in choosing the design of structural elements that provide movement of the punching device is the stepper motor location. When choosing the stepper motor orientation, it is necessary to ensure that there are no oscillations during the printing process and that the load on the belt drive is reduced during movement. When the stepper motor is located perpendicular to the shafts along which the punching device moves (option 1), oscillations occur due to the displacement of the center of gravity. This leads to a decrease in the quality of the final product. When the stepper motor is located in the lower part of the structural element (option 3), there are high loads on the lower shaft and a misalignment of the two parallel guides along which the movement occurs. Thus, the most preferred design of the structural elements and the location of the stepper motor is presented in option 2.

The developed structural elements were printed using the liquid crystal stereolithography process. The following parameters for the printing process were set: layer thickness 100 µm, number of base layers 4, curing time of 120 s per layer for the base layers, and curing time of 12 s per layer for the remaining layers. Total print time was 5 h. The resulting structural elements were integrated into a 3D printer.

The upgraded construction of the 3D printer is universal and can be used for both direct ink writing and 3D printing process using a heterophase system. The developed structural elements and the scheme of movement of the viscous ink extruder make it possible to obtain products with high accuracy, due to the absence of fluctuations in the printing process.

### 4.3. Method for Obtaining the Heterophase System based on Gelatin Microparticles in a Liquid Medium for the 3D Printing Process

To implement the 3D printing process using a modernized 3D printer construction, a method for obtaining a gelatin-based heterophase system was developed. A given amount of gelatin (3; 3.5; 4; 4.5; and 5 wt.%) is dissolved in a solution of calcium chloride with a concentration of 11 mM at a temperature of 60 °C, followed by solidification at a temperature of 4 °C for 1 h. The resulting solution is dispersed in an 11 mM calcium chloride solution in a 3:7 volume ratio using a rotor-stator homogenizer (IKA Ultra-Turrax T 25 digital) at a rotor speed of 9000 rpm for 90 s. The resulting suspension is centrifuged (Sigma 2–16 PK) at 4 °C to remove dissolved gelatin. The centrifugation process continues until a clear solution is obtained over the precipitate. Gelatin microparticles obtained as a result of repeated washing are used as a heterophase system for 3D printing.

### 4.4. Method for Obtaining Sodium Alginate Ink for 3D Printing Process Using Heterophase System

In this work, sodium alginate was used as ink. Sodium alginate is non-toxic, provides cell adhesion and proliferation, is biocompatible and biodegradable.

To implement the 3D printing process using a heterophase system, the concentration range of sodium alginate from 2 to 9 wt.% was studied. The process of obtaining solutions consisted of dissolving the alginate powder in distilled water using a rotor-stator homogenizer at a speed of 9000 rpm for 5 min, followed by centrifugation to remove air bubbles.

## Figures and Tables

**Table 1:** Examples of heterophase systems and inks for 3D printing.

Heterophase System	Ink Material	Applications	Reference	Advantages	Disadvantages
Carbopol	Bioelastomer prepolymers	Vascular tubes	[21]	High transparency of the heterophase system, which allows for the ink photopolymerization; Required rheological properties specific to Herschel–Bulkley fluid	pH sensitivity of a heterophase system; Impossibility of ionic ink crosslinking, since the ions addition to a heterophase system leads to a deterioration in its rheological properties
	Polyethylene glycol diacrylate-sodium hyaluronate	Tissue engineering	[22]		
	GelMA-gelatin-tropoelastin	Bioprinting of elastin containing bioink	[23]		
Pluronic F-127	Alginate	Tissue engineering	[24]	Biocompatibility; Required rheological properties, including shear-thinning; Ability to ionic ink crosslinking	Temperature sensitivity of a heterophase system
	Alginate	Tissue engineering, pharmaceutical testing, and organs-on-chips	[25]		
	Polydimethylsiloxane–Iron oxide nanoparticle composite	Soft magnetic helical coil actuators	[26]		
Xanthan gum methacrylate	Alginate	Tissue engineering	[27]	Biocompatibility; Required rheological properties, including shear-thinning; Ability to ionic ink crosslinking	pH sensitivity of a heterophase system
	Alginate	Tissue engineering	[28]		
Agarose	Laponite-gellan gum	Bone constructs	[29]	Biocompatibility; Required rheological properties	pH sensitivity of a heterophase system; Impossibility of ionic ink crosslinking, since the ions addition to a heterophase system leads to a deterioration in its rheological properties
	Gelatin-methacryloyl	Tissue engineering	[30]		
	Nanocomposite bioink alginate-collagen	Hierarchical fibrillar structures	[31]		

**Table 2:** Low shear rate viscosity of the heterophase system based on different gelatin concentration.

Gelatin Concentration, wt.%	Viscosity, 10^−3^ Pa∙s
3.0	5.4
3.5	10.1
4.0	14.1
4.5	22.4
5.0	32.8

**Table 3:** Low shear rate viscosity of the ink composition based on different sodium alginate concentration.

Sodium Alginate Concentration, wt.%	Viscosity, Pa∙s
2	0.8
3	1.8
4	5.7
5	8.1
6	24.6
7	44.8
8	67.1
9	118.6

**Table 4:** The dependence of the accuracy of 3D models on the concentration of the heterophase system during the 3D printing process.

Concentration of Gelatin in Heterophase System, wt.%	Accuracy, %
0	0
3	0
3.5	8.7
4	18.1
4.5	64.3
5	56.1

## Data Availability

The data that support the findings of this study are available from the corresponding author upon reasonable request.
